# A nucleotide binding rectification Brownian ratchet model for translocation of Y-family DNA polymerases

**DOI:** 10.1186/1742-4682-8-22

**Published:** 2011-06-24

**Authors:** Ping Xie

**Affiliations:** 1 Key Laboratory of Soft Matter Physics and Beijing National Laboratory for Condensed Matter Physics, Institute of Physics, Chinese Academy of Sciences, Beijing 100190, China

## Abstract

Y-family DNA polymerases are characterized by low-fidelity synthesis on undamaged DNA and ability to catalyze translesion synthesis over the damaged DNA. Their translocation along the DNA template is an important event during processive DNA synthesis. In this work we present a Brownian ratchet model for this translocation, where the directed translocation is rectified by the nucleotide binding to the polymerase. Using the model, different features of the available structures for Dpo4, Dbh and polymerase ι in binary and ternary forms can be easily explained. Other dynamic properties of the Y-family polymerases such as the fast translocation event upon dNTP binding for Dpo4 and the considerable variations of the processivity among the polymerases can also be well explained by using the model. In addition, some predicted results of the DNA synthesis rate versus the external force acting on Dpo4 and Dbh polymerases are presented. Moreover, we compare the effect of the external force on the DNA synthesis rate of the Y-family polymerase with that of the replicative DNA polymerase.

## Introduction

DNA polymerases (Pols) are enzymes to add free nucleotide to the 3' end of the newly-forming DNA strand. They play an essential role in the maintenance of genome integrity. On the basis of sequence similarity, DNA Pols can be broadly classified into A-, B-, C-, D-, X- and Y-families [1-3]. In general, most Pols in A-, B-, C-, and D-families are high-fidelity enzymes primarily involved in faithful DNA replication and in repair of replication mistake. The X-family Pols are involved in a number of DNA repair processes such as base excision repair (BER) and repair of double-strand breaks (DSBs) [4,5]. The Y-family Pols represent a number of recently identified Pols characterized by low-fidelity synthesis on undamaged DNA and the ability to bypass DNA lesions which normally block replication by members of the A-, B-, C-, D-, or X-family Pols [6-12].

The Y-family Pols are ubiquitous and are distributed among the three kingdoms of life. They include *E. coli *Pol IV (also known as DinB) [13] and Pol V (also known as UmuC) [14,15], yeast Pol η [16] and Rev1 [17], human Pols η [18], ι [19,20], κ [21] and Rev1 [22], and archaeal Dbh [23] and Dpo4 [24], etc. Although there is no detectable sequence identity with other family Pols, available crystal structures of some Y-family Pols such as Dbh [25-27], Dpo4 [28], Pol η [29,30], Pol ι [31,32], Pol κ [33,34] and Rev1 [35] reveal that they retain a catalytic core consisting of fingers, palm and thumb subdomains found in other family Pols. However, the fingers and thumb subdomains of the Y-family Pols are significantly smaller than the corresponding subdomains of the other DNA Pols. In addition to the conserved polymerase core, the Y-family Pols possess also a unique C-terminal domain termed the little finger (LF), wrist or polymerase-associated domain (PAD). In this paper we will use the acronym, LF, to denote this domain. The LF domain is the least conserved of the four domains in the Y-family Pols.

Besides the intensive structural studies of the Y-family Pols, which include the structures in apo, binary and ternary forms as well as the structures complexed with DNA substrates containing different lesions [11,25-42], a variety of biochemical assays have provided insight into the catalytic mechanism, lesion-bypassing property, processivity and fidelity of the Pols [12,43-55]. Both the biochemical and single-molecule assays for Dpo4 indicated that the binding of a nucleotide induces a fast DNA translocation event [55,56], which is consistent with the structural studies showing that, in both of the binary complexes (pre- and post-insertion), the primer terminus occupies the site where the next incoming nucleotide will bind [28,41,42]. However, the structural studies for Dbh showed that, in the pre-insertion binary complex, the templating base and the primer terminus are already positioned so that space is available for the incoming nucleotide to bind and form the ternary complex, while in the post-insertion binary complex, the DNA is located in nearly the same position on the Pol [27]. Similar to Dbh, two pre-insertion binary complexes of Pol ι showed that space is available for the incoming nucleotide to bind [32].

Recently, a Brownian ratchet model has been proposed for the translocation of the high-fidelity replicative DNA Pols, where the translocation depends on the change of the interaction of the fingers subdomain with the single-stranded DNA (ssDNA) template upon a correct incorporation [57,58]. In this work, based on the available structural, biochemical and single-molecule studies for the Y-family Pol, we modify the previous Brownian ratchet model for the replicative Pol to be applicable to the Y-family Pol, where the directed translocation is rectified by the nucleotide binding. Thus, the model can be called nucleotide binding rectification (NBR) Brownian ratchet model, which is abbreviated as the NBR model. Using the model, the observed different features of the structures for Dpo4, Dbh and Pol ι in binary and ternary forms [27,28,32,41,42] can be easily explained. Other dynamic properties for the Y-family Pols such as the considerable variations of the processivity among the Pols and the fast translocation event upon dNTP binding for Dpo4 can also be explained by using the model. In addition, some predicted results of the DNA synthesis rate versus the external force acting on Dpo4 and Dbh Pols are presented. Moreover, we compare the effect of the external force on the DNA synthesis rate of the Y-family Pol with that of the replicative Pol.

## Methods

### Brownian ratchet translocation model for replicative DNA Pols

Since the NBR model for the Y-family Pol is modified from the previous model for the replicative Pol [57-59], for convenience of reading, in this section we re-present the latter model. Briefly, the model was based on the Brownian ratchet mechanism (e.g., see [60,61]) and the directed translocation of the Pol along the template resulted from the potential change induced by dNTP incorporation. The model was built up based on two arguments.

**Figure 1:**
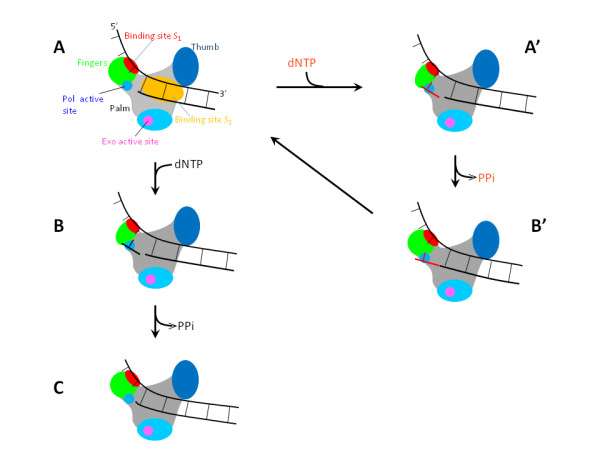
**Schematic illustrations of the translocation model for replicative DNA Pols (see text for detailed description)**. The green circles in (a), (c) and (b') denote open fingers while the green ellipses in (b) and (a') denote closed fingers.

The first argument is on the interaction between the Pol and DNA substrate. The interaction can be characterized by two DNA-binding sites on the Pol. (i) The binding site *S*_1_, which is located in the fingers subdomain (see Figure 1a), shows a high affinity for the unpaired base and/or the sugar-phosphate backbone of the ssDNA template. The presence of binding site *S*_1 _is supported by the experimental data on bacteriophage T4 DNA Pol and Klenow fragment, showing that the fingers subdomain has a high binding affinity for the ssDNA template [62-64]. (ii) The binding site *S*_2_, which is located in the palm and thumb subdomains (see Figure 1a), shows a high affinity for the double-stranded DNA (dsDNA).

The second argument is on the rotation of the fingers subdomain from open (closed) to closed (open) conformation upon the binding (release) of dNTP (pyrophosphate, PPi), which is consistent with the structural studies on bacteriophage T7 DNA Pol [65], *Taq *DNA Pol [66] and HIV-1 reverse transcriptase [67]. The closed conformation of the fingers activates the phosphodiester bond formation (or nucleotide incorporation), while the open conformation of the fingers opens the polymerase active site for nucleotide binding. Moreover, the closed fingers could potentially enhance the interactions of binding sites *S*_1 _and *S*_2 _with the DNA substrate.

Based on the two arguments, the translocation model for the replicative DNA Pol is schematically shown in Figure 1[57-59]. We begin with the binding site *S*_1 _of the Pol binding strongly to the ssDNA at the replication fork, with the binding site *S*_2 _binding to the dsDNA and no nucleotide being in the polymerase active site (Figure 1a). In this nucleotide-free state, either a matched or a mismatched dNTP can bind to the active site, although the matched dNTP has a much larger probability to bind. Thus, we consider the two cases separately. (i) First, consider a correct incorporation. The binding of a matched dNTP induces the fingers to rotate from open to closed conformations (Figure 1b). The closed conformation activates nucleotide incorporation. After the incorporation, the release of PPi induces the fingers to return to the open conformation. At the same time, the binding site *S*_1 _would bind to new nearest unpaired base (i.e., the next unpaired base) of the ssDNA template, because the previous unpaired base where the binding site *S*_1 _has just bound has disappeared due to base pairing (Figure 1c). Then, the next nucleotide-incorporation cycle will proceed. (ii) Second, consider an incorrect incorporation. We still begin with Figure 1a. The binding of a mismatched dNTP also induces the fingers to rotate from open to closed conformations, activating nucleotide incorporation (Figure 1a'). After the incorporation, the release of PPi induces the fingers to return to the open conformation. Now, although the sugar-phosphate backbone of the mismatched dNTP has been connected to the backbone of the already formed dsDNA, the mismatched base is not paired with the sterically corresponding base on the ssDNA template. Thus, the binding site *S*_1 _is still binding strongly to the same unpaired base of the ssDNA template (Figure 1b'). Thus, the polymerization cannot proceed. In other words, the polymerization becomes stalled. In Figure 1b', after the mismatched base is excised, the polymerization will proceed again (Figure 1a).

**Figure 2:**
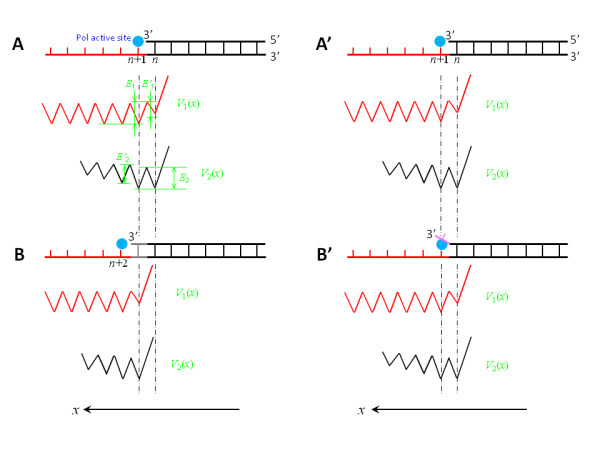
**Illustrations of the translocation model for replicative DNA Pols by using interaction potentials of binding sites *S*_1 _and *S*_2 _with ssDNA and dsDNA segments, respectively, of a DNA substrate**. (a) Top diagram shows the DNA substrate before the incorporation of the nucleotide paired with the (*n*+1)th base on the template. Potential *V*_1_(*x*) describes the interaction of the binding sites *S*_1 _with the ssDNA segment, while potential *V*_2_(*x*) describes the interaction of the binding sites *S*_2 _with the dsDNA segment. (b) The DNA substrate and potentials *V*_1_(*x*) and *V*_2_(*x*) after the incorporation of the nucleotide paired with the (*n*+1)th base on the template. (a') The DNA substrate and the potentials *V*_1_(*x*) and *V*_2_(*x*) before the incorporation of an incorrect nucleotide opposite to the (*n*+1)th base on the template, which is the same as (a). (b') The DNA substrate and potentials *V*_1_(*x*) and *V*_2_(*x*) after the incorporation of an incorrect nucleotide opposite to the (*n*+1)th base on the template.

Using potentials of the two binding sites interacting with the DNA substrate, we describe the model as follows. First, consider potential, *V*_1_(*x*), of the binding site *S*_1 _interacting with ssDNA, where position, *x*, of the Pol along the template is represented by that of its active site. Considering that the binding site *S*_1 _covers *N*_1 _bases on the ssDNA template, before the incorporation of nucleotide paired with the (*n*+1)th base (top of Figure 2a), the form of *V*_1_(*x*) is shown in Figure 2a, where *E*_1 _is the binding affinity for *N*_1 _bases of the ssDNA template while *E'*_1 _is the binding affinity for (*N*_1_-1) bases. Note that the binding affinity *E'*_1 _that corresponds to binding (*N*_1_-1) bases is smaller than *E*_1 _that corresponds to binding *N*_1 _bases. Moreover, it is implicated in the potential that the primer 3' terminus, due to the structural restriction, is not allowed to move forwards relative to the Pol when its active site is located at the primer 3' terminus. Similarly, considering that the binding site *S*_2 _covers *N*_2 _base pairs of dsDNA, before the incorporation of nucleotide paired with the (*n*+1)th base (top of Figure 2a), the potential, *V*_2_(*x*), of binding site *S*_2 _interacting with dsDNA is shown in Figure 2a. From Figure 2a, it is seen that the deepest well of the total potential, *V*(*x*) = *V*_1_(*x*) + *V*_2_(*x*), of the Pol interacting with the DNA substrate is located at position of the (*n*+1)th base before the incorporation of the nucleotide paired with the (*n*+1)th base. Thus, the Pol is now located at position of the (*n*+1)th base. After the incorporation (top of Figure 2b), the forms of *V*_1_(*x*) and *V*_2_(*x*) are shown in Figure 2b. Now, the deepest well of the total potential, *V*(*x*) = *V*_1_(*x*) + *V*_2_(*x*), is located at position of the (*n*+2)th base. Thus, the Pol would move from a shallower potential well located at position of the (*n*+1)th base to the deepest well located at position of the (*n*+2)th base.

However, after an incorrect incorporation of the nucleotide opposite to the (*n*+1)th base (see top of Figure 2b'), the forms of *V*_1_(*x*) and *V*_2_(*x*) are shown in Figure 2b', which are the same as those before the incorporation. This is because, after the incorrect incorporation, the (*n*+1)th base has not formed a base pair with the newly incorporated primer base and, thus, the Pol is still located at the position of the (*n*+1)th base, i.e., the position of the deepest well.

In this model, the translocation step occurs following the incorporation of a correct nucleotide. This is supported by the comparison of the binary (Pol-DNA) with ternary (Pol-DNA-dNTP) structures for the replicative Pol (see, e.g., [66]). Upon an incorrect incorporation, the Pol becomes stalled, which is also consistent with the experimental data [68]. For a lesion such as an abasic lesion having a weak effect on distortion of the DNA structure so that the damaged base still has a high affinity for the binding site *S*_1_, an incorporated base opposite to the lesion, which is equivalent to a mismatched base, also induces the stall of the polymerization. This is consistent with the structural observation [69]. During the stalled period, the mismatched base would be excised. Then another base opposite to the lesion site would be incorporated. Thus, the Pol cannot perform the translesion synthesis. For lesions that severely distort the DNA structure causing damaged DNA substrate not to be tolerated by the replicative Pol, e.g., with the template base being flipped out of the active site, this would preclude closing of the fingers subdomain upon nucleotide binding, as observed by Li et al. [70] for bacteriophage T7 DNA Pol complexed with a DNA template containing a *cis*-*syn *cyclobutane pyrimidine dimer. Without the activation by the closed conformation, the nucleotide incorporation cannot proceed and, thus, the Pol cannot also perform the translesion synthesis.

### Nucleotide binding rectification Brownian ratchet model for Y-family DNA Pols

The NBR model for the Y-family DNA Pol is modified from the above model for the replicative Pol. The model is also constructed based on two arguments, which are presented in the following two sections.

#### Interaction of Pol with DNA substrate

**Figure 3:**
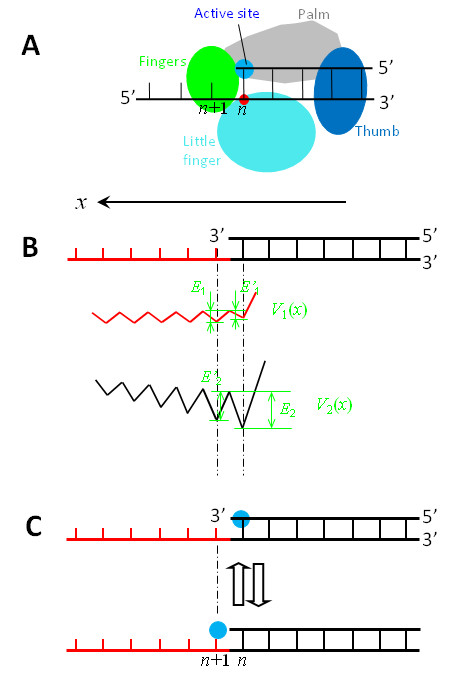
**Interaction potentials between a Y-family DNA Pol such as Dpo4, in which the active site is very close along the *x *direction to the nearest residue of the binding site *S*_2 _located in the LF domain, and a DNA substrate shown in top of (b)**. (a) Schematic diagram of the Pol complexed with the DNA substrate. (b) *V*_1_(*x*) represents the potential of the binding site *S*_1 _interacting with the ssDNA segment, while *V*_2_(*x*) represents the potential of the binding site *S*_2 _interacting with the dsDNA segment. (c) Schematic diagrams of the position of the Pol along the DNA substrate, with blue dots representing the active site.

**Figure 4:**
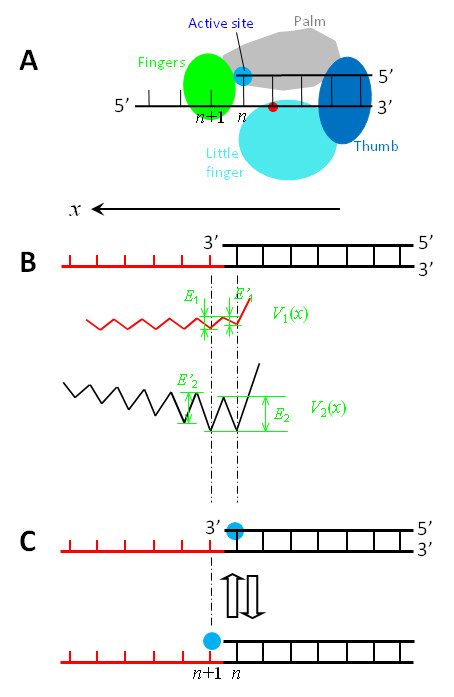
**Interaction potentials between a Y-family DNA Pol such as Dbh, in which the active site is, along the *x *direction, distanced away from (or not close to) the nearest residue of the binding site *S*_2 _located in the LF domain, and a DNA substrate shown in top of (b)**. (a) Schematic diagram of the Pol complexed with the DNA substrate. (b) *V*_1_(*x*) represents the potential of the binding site *S*_1 _interacting with the ssDNA segment, while *V*_2_(*x*) represents the potential of the binding site *S*_2 _interacting with the dsDNA segment. (c) Schematic diagrams of the position of the Pol along the DNA substrate, with blue dots representing the active site.

As in the replicative Pol (see above), the interaction of the Y-family Pol with the DNA substrate can also be characterized by two DNA-binding sites on the Pol. The binding site *S*_1 _is composed of residues located in the fingers subdomain (see Figure 3a or 4a). However, in contrast to the replicative Pol where the binding site *S*_1 _has a *high *affinity for the unpaired bases and/or the sugar-phosphate backbone of the ssDNA template, the binding site *S*_1 _in the Y-family Pol has a *very low or even no *affinity, which is consistent with the available structural studies [27,28,32,41,42]. The binding site *S*_2_, which is composed of residues located in the thumb domain and mainly in the LF domain (see Figure 3a or 4a), has a high affinity for dsDNA, which is also consistent with the available structural studies [27,28,32,41,42].

As in Figure 2, the potential *V*_1_(*x*) of the binding site *S*_1 _interacting with the ssDNA is shown in Figures 3b and 4b, with *E*_1 _denoting the binding affinity for *N*_1 _bases of the ssDNA template while *E'*_1 _the binding affinity for (*N*_1_-1) bases. However, *E'*_1 _and *E*_1 _have very small or nearly zero values.

Then, consider the potential *V*_2_(*x*) of the binding site *S*_2 _interacting with the dsDNA. Since the binding site *S*_2 _in the Y-family Pols is composed of residues located in the thumb domain and mainly in the LF domain, the form of potential *V*_2_(*x*) depends on the distance, *L*, from the active site to the nearest residue (red dots in Figures 3a and 4a) of the binding site *S*_2 _located in the LF domain along the *x *direction.

(i) For the case that the active site is very close along the *x *direction to the nearest residue of the binding site *S*_2 _located in the LF domain (see Figure 3a), as seen from the structure of Dpo4 [28,41,42], the interaction potential *V*_2_(*x*) can be simply shown in Figure 3b, where *L *= 0. If binding site *S*_2 _is considered to cover *N*_2 _base pairs of the dsDNA, *E*_2 _is the binding affinity for the sugar-phosphate backbones connecting *N*_2 _base pairs on the dsDNA while *E'*_2 _is the binding affinity for the backbones connecting only (*N*_2_-1) base pairs. Moreover, in the potential it is implicated that the primer 3' terminus, due to the structural restriction (see, e.g., [27,28,32,41,42]), is not allowed to move forwards relative to the Pol when its active site is located at the primer 3' terminus. In addition, from the Pol structures complexed with the DNA substrate, it is inferred that that the interaction between the binding site *S*_2 _and the dsDNA is via the hydrogen-bonding, van der Waals and mainly electrostatic forces. On the other hand, the interaction distance of the electrostatic force that is approximately equal to the Debye length (~ 1 nm) in solution is larger than the distance *p *= 0.34 nm between two successive base pairs. Thus, the value at maxima of *V*_2_(*x*) increases as the binding site *S*_2 _deviates away from the dsDNA segment along the *x *direction.

(ii) For the case that the active site is, along the *x *direction, distanced away from (or not close to) the nearest residue of the binding site *S*_2 _located in the LF domain (Figure 4a), as evidently seen from the structure of Dbh [27], the interaction potential *V*_2_(*x*) can be simply shown in Figure 4b, where we take *L *= 1 bp. From available structures of the binary and ternary complex for Pol ι [31,32], it is also noted that, if the active site is positioned opposite to the first unpaired base on the template, the first unpaired base is distanced by *L *= 1 bp away from the nearest residue of the binding site *S*_2 _located in the LF domain. Thus, the interaction potential *V*_2_(*x*) for Pol ι also has the form of Figure 4b rather than that of Figure 3b. Similarly, from the available structure of the ternary complex for Pol η [30], we infer that the interaction potential *V*_2_(*x*) for Pol η also has the form of Figure 4b.

From Figure 3b it is seen that, when the active site is positioned at the *n*th base pair (top of Figure 3c), the affinity of the Pol for the DNA substrate is *E_n _*= *E'*_1 _+ *E*_2_; while when the active site is positioned at the (*n*+1)th base (bottom of Figure 3c), the affinity is *E*_*n*+1 _= *E*_1 _+ *E'*_2_. Since *E'*_1 _and *E*_1 _are much smaller than *E'*_2 _and *E*_2 _and *E*_2 _>*E'*_2_, it is expected that *E_n _*>*E*_*n*+1_. Similarly, from Figure 4b it is seen that, when the active site is positioned at the *n*th base pair (top of Figure 4c), the affinity of the Pol for the DNA substrate is *E_n _*= *E'*_1 _+ *E*_2_; while when the active site is positioned at the (*n*+1)th base (bottom of Figure 4c), the affinity is *E*_*n*+1 _= *E*_1 _+ *E*_2_. Since *E'*_1 _and *E*_1 _are much smaller than *E*_2_, it is expected that *E*_*n*+1 _is slightly larger than (or nearly equal to) *E_n_*. Moreover, from both Figure 3 and 4 it is noted that, when the active site is positioned at the (*n*+1)th base, the jumping of the Pol from the (*n*+1)th site to the (*n*+2)th site is required to overcome a larger energy barrier than the backward jumping to the *n*th site. For approximation, we do not consider the jumping to the (*n*+2)th site in this work.

#### The binding of dNTP induces a slight conformational change, enhancing the interaction of the Pol with DNA substrate

As evidenced from the FRET experimental data [56], it is argued that the dNTP binding involves (at least) two substeps, E · DNA + dNTP → E · DNA · dNTP → E* · DNA · dNTP, where E represents the DNA Pol. The transition from the unactivated E · DNA · dNTP ternary complex to activated E* · DNA · dNTP ternary complex induces a slight conformational change of the Pol, enhancing its interactions with both the DNA substrate and dNTP. Similarly, the PPi releasing also involves (at least) two substeps, E* · DNA · PPi → E · DNA · PPi → E · DNA + PPi, where the transition from the activated E* · DNA · PPi ternary complex to unactivated E · DNA · PPi ternary complex results in a reverse slight conformational change of the Pol, reducing its interactions with both the DNA substrate and PPi.

Since in the activated E* · DNA · dNTP (or E* · DNA · PPi) complex the Pol has a stronger interaction with DNA substrate and nucleotide than in the unactivated E · DNA · dNTP (E · DNA · PPi) complex, for simplicity of analysis, it is considered that in the activated state the Pol is unable to move relative to the DNA substrate and the dNTP or PPi bound to the active site has a negligible probability to release.

#### Model for Pol translocation

**Figure 5:**
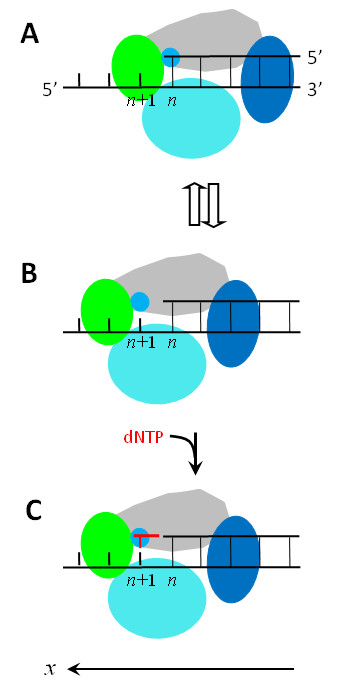
**Schematic illustrations of the nucleotide binding rectification Brownian ratchet model for the Y-family DNA Pol translocating along DNA substrate (see text for detailed description)**.

Using potentials *V*_1_(*x*) and *V*_2_(*x*) (Figures 3 and 4), the NBR model for the Y-family Pol translocating along DNA substrate is schematically shown in Figure 5.

We begin with the Pol positioned at the *n*th site (Figure 5a), just after the incorporation of a nucleotide. In Figure 5a, the active site is occupied by the primer 3'-terminus, which sterically prevents a dNTP from binding to the active site. Due to the thermal noise, the Pol in this nucleotide-free state can jump from the *n*th site to the (*n*+1)th site (from Figure 5a to 5b) and vice verse (from Figure 5b to 5a). For the case that the active site is very close along the *x *direction to the nearest residue of the binding site *S*_2 _located in the LF domain (Figure 3a), *E_n _*>*E*_*n*+1 _(see above). Thus, the Pol in the binary E · DNA state stays most of the time at the *n*th site (Figure 5a), as will be shown in the Results, which is consistent with the availably resolved binary E · DNA structure for Dpo4 [28,41,42]. For the case that the active site is, along the *x *direction, distanced away from (or not close to) the nearest residue of the binding site *S*_2 _located in the LF domain (Figure 4a), *E*_*n*+1 _is slightly larger than (or nearly equal to) *E_n _*(see above). Thus, the Pol in the binary E · DNA state shows slightly larger (or nearly equal) probability to stay at the (*n*+1)th site (Figure 5b) than (or to) that at the *n*th site (Figure 5a), implying that the binary E · DNA structure for this case would be observed to be either at the (*n*+1)th site or at the *n*th site. This is consistent with the observations that the pre-insertion binary E · DNA structures for Dbh [27] and Pol ι [32] showed that their active sites are at the (*n*+1)th site, while the post-insertion binary E · DNA structure for Dbh [27] showed that the active site is at the *n*th site.

When the Pol jumps to the (*n*+1)th site, since the active site is nucleotide free, a dNTP becomes able to bind to it, as shown in Figure 5b that is equivalent to the state shown at bottom of Figure 3c or Figure 4c. Consider that the dNTP binds to the active site during the period when the Pol stays at the (*n*+1)th site (Figure 5c). Due to the structural restriction (see, e.g., [27,28,32,41,42]), the occupation of the active site by the dNTP sterically prevents the Pol from moving backwards to the *n*th site unless the dNTP is dissociated, which is consistent with the available structures showing that the active site of the Pols such as Dbh, Dpo4, Pol ι, Pol η, Pol κ and Rev1 in ternary forms is at the (*n*+1)th site [11,27,28,30,32,34,35,41,42]. Then, the transition from the unactivated ternary complex E · DNA dNTP to the activated E* · DNA dNTP complex enhances the interactions of the Pol with the DNA substrate and with the dNTP, thus preventing both the DNA substrate and the dNTP from dissociating from the Pol.

After the phosphodiester bond formation and then the release of PPi, except that the dsDNA segment is elongated by one base pair and the Pol has moved forwards by one base pair, the Pol-DNA complex returns to the state shown in Figure 5a. Correspondingly, the potentials *V*_1_(*x*) and *V*_2_(*x*) in Figure 3b and in Figure 4b are shifted by one base pair along the *x *direction. Then, the next round of the nucleotide incorporation would proceed continuously.

#### Equations for Pol motion

Consider the movement of Pol relative to the DNA substrate in two dimensions. One is along the DNA, which is represented by the *x *axis, as shown in Figures 3, 4, and 5. The other one is along the *r *axis that is perpendicular to the *x *axis. Then, the movement equations can be written in the following Langevin forms(1a)(1b)

Here the potential *U*(*x,r*) can be written as *U*(*x,r*) = *V*(*x*)[2exp (-*r*/*r_d_*) - exp (-2*r*/*r_d_*)], with *V*(*x*) = *V*_1_(*x*) + *V*_2_(*x*) + *V*_0_, where *V*_1_(*x*) and *V*_2_(*x*) have the forms shown in Figures 3b and 4b, and *V*_0 _≡-*E*_0 _< 0 results from the fact that the electrostatic interaction distance of the Pol with the DNA in solution is larger than the distance between two successive base pairs. The magnitude of *V*(*x*) is defined as follows: its minimum value at the *n*th site is - (*E_n _*+ *E*_0_), while at the (*n*+1)th site the value is - (*E*_*n*+1 _+ *E*_0_). The term [2 exp (-*r*/*r_d_*) - exp (-2*r*/*r_d_*)], which has the Morse form, denotes the potential change along the *r *direction, with 2*r_d _*= 1 nm (the Debye length) characterizing the interaction distance. The parameter Γ is the frictional drag coefficient on the Pol and ξ*_i _*(*t*) (*i *= *x*, *r*) is the fluctuating Langevin force with 〈*ξ*_*i *_(*t*)〉 = 0 and 〈*ξ*_*i *_(*t*)*ξ*_*j *_(*t*')〉 = 2*k*_*B *_*T*Γ*δ*_*ij *_*δ*(*t *- *t*'). The drag coefficient is calculated by Γ = 6*πηR*, where η is the viscosity of the aqueous medium and the Pol is approximated as a sphere with radius *R *= 5 nm. As the previous experiment showed that the viscosity of the aqueous cytoplasm does not differ from water [71], we take the viscosity of aqueous to be the same as that of water in the calculation, i.e., *η *= 0.01 g cm^-1 ^s^-1^, which gives Γ = 9.4 × 10^-11 ^kg s^-1^. Moreover, the effect of the viscosity variation on the results will be discussed.

## Results

### Processivity of the Y-family Pol

To study the processivity of the Y-family Pol, we determine the dissociation probability of the Pol from the DNA substrate during one cycle of nucleotide incorporation. To this end, we calculate the mean dissociation time, *T_d_*, of the Pol from the DNA substrate.

First, we consider the motion with the Pol fixed at one potential well (e.g., the potential well at the *n*th site) along the *x *direction. Then, the potential *U*(*x,r*) in Eq. (1b) becomes: *W*(*r*) = -*E_r _*[2 exp (-*r*/*r_d_*) - exp (-2*r*/*r_d_*)], where the depth of the potential well is *E_r _*= *E_n _*+ *E*_0_. If it is considered that the Pol is dissociated from its DNA substrate when it moves away from the DNA substrate by a distance of *r *= *L*, the mean dissociation time *T_d_*, i.e., the mean time for the Pol to move from *r *= 0 to *r *= *L*, can be obtained by [72](2)

where D = *K_B_T*/Γ. From Eq. (2) we have(3)

where it is seen that *T_d _*is proportional to the viscosity *η*.

The dissociation probability per unit time, *P_d_*, of the Pol from the DNA substrate is calculated by(4)

It is noted from Eqs. (3) and (4) that *P_d _*is inversely proportional to the viscosity *η*.

Based on the model, only during the time period, *T_p*1*_*, after transition to the unactivated E · DNA PPi ternary complex but before the dNTP binding and during the time period, *T*_*p*2_, after the dNTP binding but before transition to the activated E* · DNA dNTP ternary complex, can the Pol be dissociated from the DNA substrate. During the time period *T*_*p*1_, the Pol can jump between the well at the *n*th site and the well at the (*n*+1)th site along the *x *direction. As our results show (see additional file 1), the dissociation probability *P_d1 _*during this time period *T*_*p*1 _is approximately only dependent on the value of  (*m *= *n *or *n *+ 1), with , where *C *= 1 ~ 2 and  represents *P_d _*given by Eqs. (3) and (4) but with  (*m *= *n *if *E_n _*>*E*_*n*+1_, *m *= *n *+ 1 if *E_n _*<*E*_*n*+1_). During the time period *T*_*p*2_, the Pol is positioned at the (*n*+1)th site and the dissociation probability *P_d2 _*is calculated by , where  represents *P_d _*given by Eqs. (3) and (4) but with .

For the Pol such as Dpo4, in which the active site is very close along the *x *direction to the nearest residue of the binding site *S*_2 _located in the LF domain, since *E_n _*>*E*_*n*+1_, it is noted from Eqs. (3) and (4) that . Moreover, it is known that *T*_*p*1 _<*T*_*p*2 _at saturating concentrations of dNTP for Dpo4 [56]. Thus, we have . The mean number of incorporated nucleotides for one binding event of the Pol with the DNA substrate, which characterizes the polymerization processivity, is calculated by(5)

For the Pol such as Dbh, Pol ι and Pol η, in which the active site is, along the *x *direction, distanced away from (or not close to) the nearest residue of the binding site *S*_2 _located in the LF domain, since *E*_*n*+1 _≥ *E_n_*, we have . Thus, the mean number of incorporated nucleotides for one binding event is calculated by(6)

From Eqs. (5) and (6), it is seen that, whether *E_n _*>*E*_*n*+1 _or *E_n _*≤ *E*_*n*+1_, the polymerization processivity is mainly determined by the binding affinity, *E*_*n*+1_, of the Pol at the (*n*+1)th site along the DNA substrate. Moreover, it is noted that *N_p _*is proportional to the viscosity *η*.

**Figure 6:**
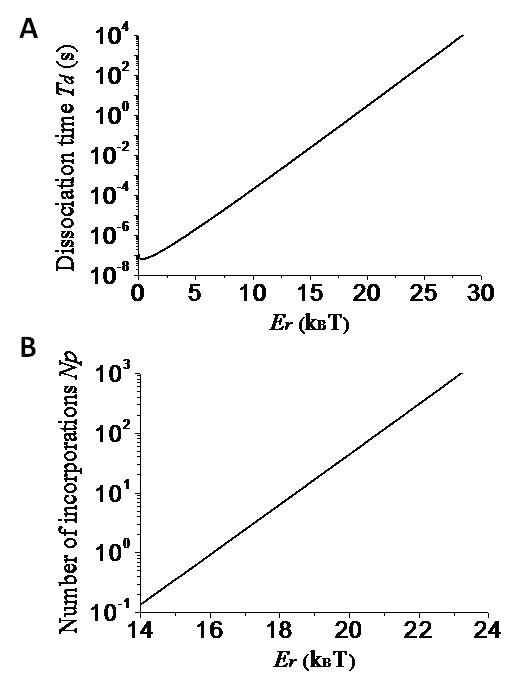
**Results for processivity of the Y-family Pol**. (a) Mean dissociation time *T_d _*of the Pol from the DNA substrate versus the binding affinity *E_r _*between them. (b) Mean number of processive incorporation cycles *N_p _*versus *E_r_*.

Using Eq. (3), the calculated results of the mean dissociation time *T_d _*versus *E_r _*are shown in Figure 6a, where we take *L *= 5 nm that is larger than the interaction distance 2*r_d _*= 1 nm. It is seen that *T_d _*increases significantly with the increase of *E_r_*. With results of Figure 6a and using Eqs. (4) and (5), the calculated results of *N_p _*versus *E_r _*are shown in Figure 6b, where we take *T*_*p*2 _= 0.065 s that is consistent with the experimental data of transition rate of 15.3 s^-1 ^for Dpo4 [56]. It is seen that, when *N_p _*= 10 ~ 100 that is consistent with the experimental data [48], *E_r ≈ _*18.5*k_B_T *~ 20.8*k_B_T*. Now, from this value of , we estimate values of *E*_*n*+1 _and *E_n_*. Taking a conservative value of *E*_0 _= *E_r_*/5 = 3.9*k_B_T*, we estimate that the value of *E*_*n*+1 _is at most about 17*k_B_T*. For reasonable value of *E_n_*-*E*_*n*+1 _= 3*k_B_T *~ 5*k_B_T*, we estimate that the value of *E_n _*is at most about 20*k_B_T *~ 22*k_B_T*.

In addition, from Figure 6b it is interesting to see that, if *E_r _*is decreased from 20.8*k_B_T *to a value below 16*k_B_T*, *N_p _*is decreased from about 100 to a value smaller than 1. This implies that only an about 5*k_B_T *decrease in the binding affinity can induce the processive polymerization of a hundred nucleotides to a distributive polymerization. From this result, it is also concluded that the considerable variations of the processivity among Y-family Pols result mainly from slight changes in their binding affinities for the DNA substrate. Moreover, since the LF domain is the least conserved of the four domains in the Y-family Pols, the slight differences in the binding affinity of different Pols are mainly due to different interaction strengths of the LF domain with the DNA. For example, comparison of the LF structure of Dpo4 with that of Dbh showed that the DNA-contacting surface in LF domain of Dpo4 is slightly more positively charged than Dbh, and, correspondingly, Dpo4 is much more processive than Dbh [48]. Since the LF domain has a large binding affinity for the DNA, it is expected from Figure 6a that the deletion of the LF domain will significantly reduce the association time of the Pol with the DNA, thus resulting in much less active than the full-length Pol. This is also consistent with the experimental data [28].

### Moving time of the Y-family Pol

Now, we study the moving time from the *n*th site to the (*n*+1)th site and vice verse during the time period after the incorporation of the *n*th base but before the dNTP binding. To this end, we can consider the motion only along the *x *direction and the potential *U*(*x,r*) in Eq. (1a) becomes *V*(*x*). Thus, the mean moving time, *T*_*n*→(*n*+1)_, i.e., the mean first-passage time for the Pol to move from the *n*th site at position *x *= 0 to the next (*n*+1)th site at position *x *= *p *= 2*l *= 0.34 nm can be approximately calculated by , which is similar to Eq. (2). From the integral, we have(7)

The mean moving time, *T*_(*n*+1)→ *n*_, from the (*n*+1)th site to the *n*th site also has the form of Eq. (7) but with *E_n _*and *E*_*n*+1 _being exchanged with each other. From Eq. (7) and D = *K_B_T*/Γ, it is noted that the mean moving time is proportional to the viscosity *η*.

**Figure 7:**
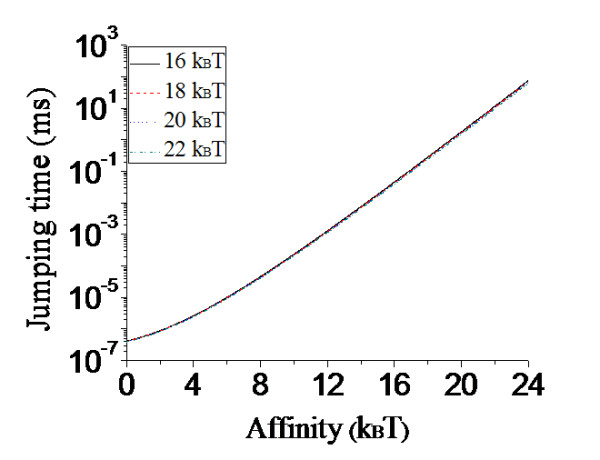
**Results of the mean time *T*_*n*→(*n*+1) _(*T*_(*n*+1)→*n*_) for the Y-family Pol to move from the *n*th ((*n*+1)th) site to the (*n*+1)th (*n*th) site versus *E_n _*(*E*_*n*+1_) for different values of *E*_*n*+1 _(*E_n_*) (indicated in the figure) before dNTP binding to the active site**. Note that the four curves of *T*_*n*→(*n*+1) _(*T*_(*n*+1)→*n*_) versus *E_n _*(*E*_*n*+1_) for different values of *E*_*n*+1 _(*E_n_*) are nearly coincident.

Using Eq. (7), the calculated results of the mean moving time *T*_*n*→(*n*+1) _(*T*_(*n*+1)→ *n*_) versus *E_n _*(*E*_*n*+1_) for different values of *E*_*n*+1 _(*E_n_*) are shown in Figure 7. As expected, *T*_*n*→(*n*+1) _increases significantly with the increase of *E_n _*but is insensitive to the variation of *E*_*n*+1_, while *T*_(*n*+1)→ *n *_increases significantly with the increase of *E*_*n*+1 _but is insensitive to the variation of *E_n_*. It is seen from Figure 7 that, even for the value of *E_n _*= 20*k_B_T *~ 22*k_B_T *for Dpo4 (see the above section), the mean moving time *T*_*n*→(*n*+1) _is only about 2 ~ 10 ms. For the value of *E*_*n*+1 _= 17*k_B_T *for Dpo4 (see the above section), *T*_(*n*+1)→ *n *_is only about 0.1 ms. These results indicated that, after the incorporation of the *n*th base and before the dNTP binding, Dpo4 would jump between the *n*th site and the (*n*+1)th site with a high frequency. Thus, within the time resolution used in the FRET experiment [56], this highly frequent jumping between the two positions could not be detected. Moreover, as will be shown in the following section, Dpo4 would stay most probably at the *n*th site (Figure 5a). Thus, the resolved structure is most probably in the state with Dpo4 active site being located at the *n*th site and the FRET data shows a rapid translocation event for Dpo4 relative to the DNA substrate upon the adding of dNTP, which is consistent with the experimental data [41,42,56].

### Effect of external force on DNA-synthesis rate of the Y-family Pol

In the NBR model for the Y-family Pol, after the incorporation of the *n*th base and before the dNTP binding, the active site jumps between the *n*th site and the (*n*+1)th site. As noted from Eq. (7), for *E_n _*> > 1 and *E*_*n*+1 _> > 1, the ratio of the time, *T_n_*, for the active site to position at the *n*th site over the time, *T*_*n*+1_, to position at the (*n*+1)th site approximately has the form(8)

For Dpo4, since *E_n _*>*E*_*n*+1_, it is thus noted from Eq. (8) that the active site has a much larger probability to stay at the *n*th site than to stay at the (*n*+1)th site. For the value of *E_n_*-*E*_*n*+1 _= 3*k_B_T *~ 5*k_B_T*, *T_n_*/*T*_*n*+1 _≈ 20 ~ 100. For Dbh, Pol ι and Pol η, *E*_*n*+1 _is slightly larger than (or nearly equal to) *E_n_*. From Eq. (8) it is noted that the Pols show slightly larger (or nearly equal) probability to stay at the (*n*+1)th site than (or to) that at the *n*th site.

Consider an external force, *F*, acting on the Pol bound to a fixed DNA substrate, where *F *is defined as positive when it points towards the -*x *direction. The experiment can be realized by using the optical trapping method, with a micro-bead linked to the residues on the palm subdomain or LF domain of the Pol. The linked residues on the Pol should be far away from the active site, thus the external force having no effect on the polymerase activity of the active site. Under the external force *F*, the depth of potential well at the *n*th site changes from *E_n _*to *E_n _*+ *Fp*/2, while the depth at the (*n*+1)th site changes from *E*_*n*+1 _to *E*_*n*+1_-*Fp*/2. Thus, Eq. (8) becomes(9)

Based on the model, only when the active site is positioned at the (*n*+1)th site can the dNTP bind to the active site, i.e., only during the time period *T*_*n*+1 _can the dNTP bind to the active site. Thus, based on Eq. (9), the dNTP-binding rate, *k_b_*(*F*), versus the external force *F *has the form(10)

where  denotes the dNTP-binding rate under no external force. From Eq. (10), the DNA-synthesis rate, *k*, is calculated by(11)

where *k_c _*is the dNTP-incorporation rate at saturating dNTP concentration and . It is noted from Eqs. (8) - (11) that the DNA-synthesis rate *k *is independent of the viscosity *η*.

**Figure 8:**
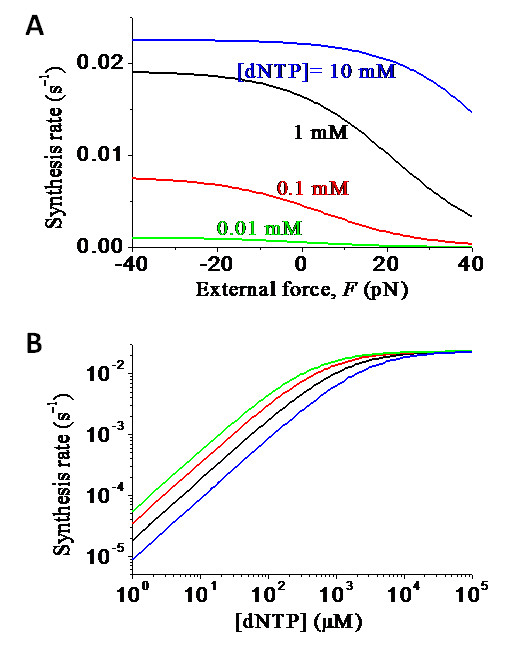
**Predicted results of DNA-synthesis rate *k*(*F*) under the effect of the external force *F *for Dbh**. (a) DNA-synthesis rate *k*(*F*) versus *F *for different values of [dNTP]. (b) DNA-synthesis rate *k*(*F*) versus [dNTP] for different values of *F*, with curves from upper to lower corresponding to *F *= 1 pN, 10 pN, 20 pN and 30 pN, respectively.

**Figure 9:**
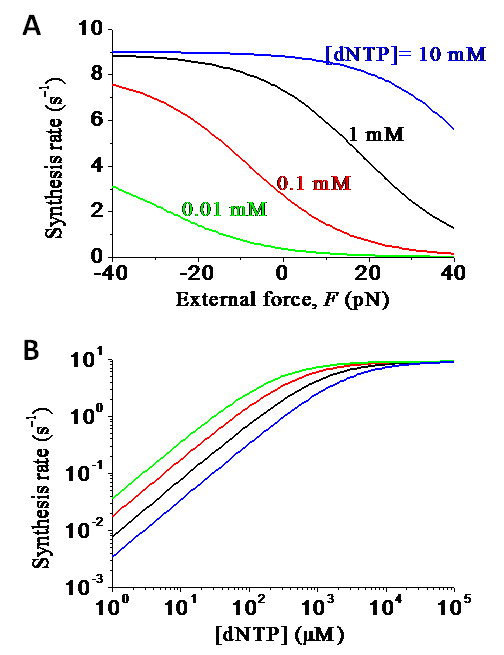
**Predicted results of DNA-synthesis rate *k*(*F*) under the effect of the external force *F *for Dpo4**. (a) DNA-synthesis rate *k*(*F*) versus *F *for different values of [dNTP]. (b) DNA-synthesis rate *k*(*F*) versus [dNTP] for different values of *F*, with curves from upper to lower corresponding to *F *= 1 pN, 10 pN, 20 pN and 30 pN, respectively.

Now, we use Eq. (11) to make some predicted results. From the experimental data, we have *k_c _*= 2.3 × 10^-2 ^s^-1 ^and  = 0.4 mM for Dbh [54]. As discussed before, we take *E*_*n*+1 _≈ *E_n _*for Dbh. Using Eq. (11), we calculate the DNA-synthesis rate *k *versus *F *for different values of [dNTP], with the results shown in Figure 8a, and 8k versus [dNTP] for different values of *F*, with the results shown in Figure 8b. For Dpo4, *k_c _*= 9 s^-1 ^and  = 230 μM [44]. Moreover, we take *E*_*n*_-*E*_*n*+1 _= 3*k_B_T *for Dpo4. The results of *k *versus *F *for different values of [dNTP] and *k *versus [dNTP] for different values of *F *are shown in Figures 9a and 9b, respectively. By comparing Figure 8a with Figure 9a, it is seen that, in the range of *F *= -20 ~ 20 pN and [dNTP] ≤ 1 mM, the external force *F *has more significant effect on the DNA-synthesis rate *k *of Dpo4 than on that of Dbh.

### Comparison of the effect of external force on dNTP-binding rate of the Y-family Pol with that of the replicative Pol

Based on the model for the replicative Pol (Figure 2) and the modified model for the Y-family Pol (Figures 3, 4, and 5), the dNTP-binding rate *k_b_*(*F*) versus the external force *F *satisfies Eq. (10), where *E_n_*-*E*_*n*+1 _= *E'*_1_-*E*_1 _< 0 for the replicative Pol (see Figure 2) while *E_n_*-*E*_*n*+1 _≥ 0 for the Y-family Pol. For the replicative Pol, *E'*_1_-*E*_1 _represents the binding affinity of binding site *S*_1 _for one base of the ssDNA template (or the binding affinity of the binding site *S*_1 _residue that is closest to the palm subdomain for ssDNA), and it is estimated that *E'*_1_-*E*_1 _= -5*k_B_T *~ -3*k_B_T*. As mentioned before, for Dbh, Pol ι and Pol η, *E_n_*-*E*_*n*+1 _≈ 0, while for Dpo4, *E_n_*-*E*_*n*+1 _= 3*k_B_T *~5*k_B_T*.

**Figure 10:**
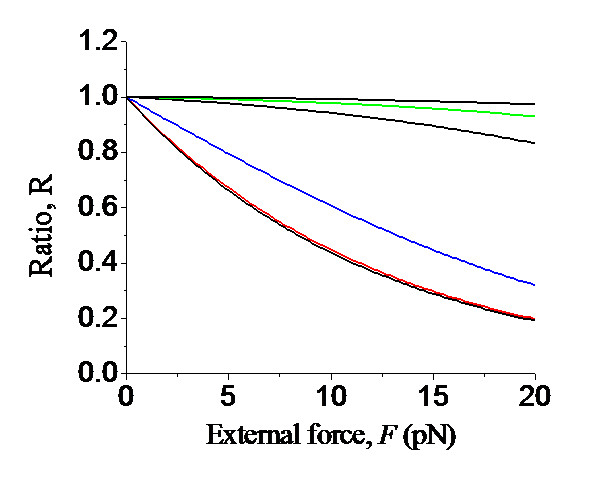
**Predicted results of ratio, , versus the external backward force *F *acting on the Pol for different values of *E_n_*-*E*_*n*+1_, where *k_b_*(*F*) is the dNTP-binding rate under effect of the external force *F *and  is the dNTP-binding rate under no external force**. Curves from upper to lower are for *E_n_*-*E*_*n*+1 _= -5*k_B_T*, -4*k_B_T*, -3*k_B_T*, 0, 3*k_B_T *and 5*k_B_T*, where *E_n_*-*E*_*n*+1 _= -5*k_B_T*, -4*k_B_T*, -3*k_B_T *correspond to replicative Pols, *E_n_*-*E*_*n*+1 _= 0 corresponds to Dbh, Pol ι and Pol η and *E_n_*-*E*_*n*+1 _= 3*k_B_T *and 5*k_B_T *correspond to Dpo4.

Using Eq. (10), the calculated results of ratio  versus the external backward force *F *acting on the Pol for different values of *E_n_*-*E*_*n*+1 _are shown in Figure 10. It is seen that, in the range of *F *< 20 pN, with the increase of *F *the ratio *R *only decreases slightly for the replicative Pol whereas the ratio *R *decreases greatly for the Y-family Pol. In other words, the external backward force has much more effect on the the dNTP-binding rate for the Y-family Pol than for the replicative Pol.

The biological implication of these different characteristics between the Y-family and replicative Pols might be imagined as follows. At the replication fork, the replicative Pol generally feels a backward force by the front DNA helicase [73]. Thus, the slight effect of the backward force on the DNA synthesis rate by the replicative Pols is purposed to have little impact on the DNA replication. However, when the replicative Pol becomes stall at the lesion site, since the front helicase is still unwinding the dsDNA, the Pol would not feel a backward force now. Thus, when the relicative Pol is replaced by the Y-family Pol, the latter would also not feel a backward force at the lesion site. After bypass the lesion site, the Y-family Pol would continue to make processive DNA synthesis and, if the Y-family Pol catches up with the helicase, the backward force induced by the front helicase would greatly reduce the DNA synthesis rate, thus enhancing the probability of the Y-family Pol to dissociate from the DNA substrate or the probability of the Y-family Pol to be replaced by the replicative Pol.

### The Y-family Pol can easily bypass a mismatched base pair or a lesion site

In this section, we will show how the Pol that uses the NBR mechanism for translocation can easily bypass a mismatched base pair or a lesion site. As an example, we will use Dpo4 to illustrate this bypass ability, in which the active site is very close along the *x *direction to the nearest residue of the binding site *S*_2 _located in the LF domain.

**Figure 11:**
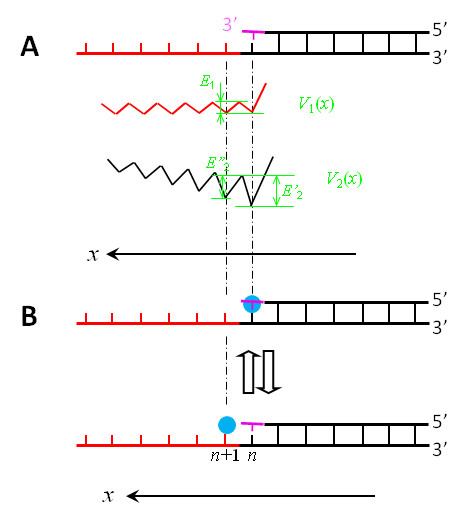
**Interaction potentials between a Y-family DNA Pol and a DNA substrate with a mismatched base pair at the *n*th site shown in top of (a)**. For clarity, the mismatched base is drawn in pink. (a) *V*_1_(*x*) represents the potential of the binding site *S*_1 _interacting with the DNA substrate, while *V*_2_(*x*) represents the potential of the binding site *S*_2 _interacting with the DNA substrate. (b) Schematic diagrams of the position of the Pol along the DNA substrate, with blue dots representing the active site.

As shown in Figure 11, consider that a mismatched base is incorporated at the *n*th site. Then, the interaction potential *V*_1_(*x*) of the binding site *S*_1 _with the ssDNA template and the potential *V*_2_(*x*) of the binding site *S*_2 _with the dsDNA segment are shown in Figure 11a. Here, *E*_1 _is the binding affinity of the binding site *S*_1 _for *N*_1 _bases of the ssDNA template that the binding site *S*_1 _can cover, *E'*_2 _is the binding affinity of the binding site *S*_2 _for the backbones connecting (*N*_1_-1) base pairs on the dsDNA, and *E''*_2 _is the binding affinity of the binding site *S*_2 _for the backbones connecting (*N*_1_-2) base pairs. Thus, when the active site is positioned at the *n*th site (top of Figure 11b), the affinity of the Pol for the DNA substrate is *E_n _*= *E*_1 _+ *E'*_2_; while when the active site is positioned at the (*n*+1)th site (bottom of Figure 11b), the affinity is *E*_*n*+1 _= *E*_1 _+ *E''*_2_. Note that the binding affinity *E''*_2 _that corresponds to binding (*N*_1_-2) base pairs is smaller than *E'*_2 _that corresponds to binding (*N*_1_-1) base pairs.

Now we compare the case of a matched incorporation at the *n*th site (Figure 3) with the case of a mismatched incorporation (Figure 11). Since *E'*_1 _and *E*_1 _are much smaller than *E'*_2 _and *E*_2_, we have , where  and  represent *E_n _*for cases of matched incorporation and mismatched incorporation, respectively. Thus, from the results shown in Figure 7, it is seen that the mean moving time *T*_*n*→(*n*+1) _for the case of mismatched incorporation is shorter than the case of matched incorporation. Moreover, it is noted that the value of  is close to that of , since both value of *E'*_2_-*E''*_2 _and that of *E*_2_-*E'*_2 _correspond to the affinity of the binding site *S*_2 _for the backbones connecting one base pair on the dsDNA. Thus, from Eq. (8), the ratio (*T*_*n*_/*T*_*n*+1_)^(*mismatch*) ^is close to (*T*_*n*_/*T*_*n*+1_)^(*match*)^. As a result, we conclude that the Pol that uses the NBR mechanism for translocation has nearly the same rate to bypass a mismatched base pair as that to bypass a matched base pair. Similarly, for the case of an abasic lesion located at the *n*th site, the interaction potential *V*_2_(*x*) is the same as shown Figure 11a. Thus, as in the case of a mismatched base, the Pol that uses the NBR mechanism for translocation can also easily bypass the lesion site.

However, since , it is noted from Figure 6a that the dissociation probability near the lesion site is larger than that in the absence of the lesion or mismatched base, which is in agreement with the experimental data for Dpo4 [52]. On the hand, it is seen from Figure 6b that, when there is no lesion or no mismatched base, for *N_p _*= 10 that corresponds to the case of Dbh [48]*E_r _*= 18.5*k_B_T*, while for *N_p _*= 100 that corresponds to the case of Dpo4 [48]*E_r _*= 20.8*k_B_T*. From the value of *E*_1_-*E'*_1 _= 3*k_B_T *~ 5*k_B_T *(see above), we infer that *E'*_1_-*E''*_1 _≈ 3*k_B_T *~ 5*k_B_T*. Thus, at the lesion site, for the case of Dbh with *E_r _*= 13.5*k_B_T *~ 15.5*k_B_T*, we have *N_p _*≈ 0.08 ~ 0.6 from Figure 6b; for the case of Dpo4 with *E_r _*= 15.8*k_B_T *~ 17.8*k_B_T*, we have *N_p _*≈ 0.8 ~ 5.2 from Figure 6b. This implies that, at the lesion site, Dbh is prone to dissociate from the DNA substrate while Dpo4 is not easily to dissociate. In other words, Dpo4 can bypass the lesion site, yet Dbh does so with a much lower efficiency. These are consistent with the experimental data [24,48,50,52]. Moreover, as the different lesion-bypassing abilities of the two Pols, Dpo4 and Dbh, are due to the different binding affinities, which in turn result mainly from the different interaction strengths of the LF domain with the DNA, it is inferred that, by interchanging the LF domains, the lesion-bypassing abilities of the two Pols will be exchanged. This is also consistent with the experimental data of Boudsocq et al. [48].

Similarly, we can easily show that the Pols such as Dbh and Pol ι, in which the active site is, along the *x *direction, not close to the nearest residue of the binding site *S*_2 _located in the LF domain, can also easily bypass the lesion site. Thus, we conclude that, although different values of distance *L *give different translocation features, all the Y-family Pols that use the NBR mechanism for translocation can easily bypass the lesion site, thus performing the translesion synthesis. By contrast, the replicative Pols that use other Brownian ratchet mechanism for translocation cannot bypass the lesion site and is thus unable to perform the translesion synthesis (see Figures 1 and 2).

## Discussion

The explanation of the lesion-bypassing ability by a Y-family Pol in this work is focused only on the translocation activity of the active site from the position opposite to the lesion site to the next position. In fact, the lesion-bypassing ability is dictated by two activities. One is the translocation activity while the other one is the catalytic activity of the phosphodiester bond formation. The latter activity determines that rates of nucleotide incorporation opposite to the lesion site and one position downstream from the lesion site are slower than those at other sites [46]. Moreover, since the latter activity is determined by the structure of the catalytic core, single amino acid substitutions within the active site, palm or fingers subdomains can also have a profound effect on the ability of the enzyme to perform translesion synthesis [74,75].

Distinct translocation features among the Y-family Pols in the NBR model depend on the distance *L *from the active site to the nearest residue of the binding site *S*_2 _located in the LF domain. It is important to note that this distance *L *in the model corresponds to the structure of the Pol only after binding to its DNA substrate. Although available structures showed a significant conformational change in the LF domain of the apo-Dpo4 upon binding to the DNA substrate [42], only a little change has been observed between the Pol complexed with the DNA substrate alone and that with both the DNA substrate and the dNTP nucleotide. Thus, the large conformational change in the LF domain of the Pol upon binding to the DNA substrate has no effect on the conclusion of the current work which is involved only with the structures of the Pol either complexed with the DNA substrate alone or complexed with both the DNA substrate and the dNTP nucleotide.

### Further comments on the NBR translocation model

In the NBR model (Figure 5), it has been implicitly considered that the Pol has a rigid structure, i.e., different domains have been considered to be linked rigidly. In fact, the residues linking different domains may behave elastically. For example, for Dpo4, considering an elastic link between the palm and thumb domains. Upon nucleotide binding, after the active site, together with the fingers, palm and LF domains, move from the *n*th site to the (*n*+1)th site, the thumb domain may not move simultaneously due to the elastic link between the palm and thumb domains, i.e., the thumb contacts with the DNA may fluctuate by the thermal noise between pre- and post-translocation positions with nearly equal probability. This gives an explanation to the available structural data for Dpo4 showing that, upon nucleotide binding, the LF contacts with the DNA shift by one base pair but the thumb contacts do not shift simultaneously [41].

### Potential implication of binding site S_1 _in the induced-fit mechanism

The strong interaction of the binding site *S*_1 _with the unpaired base on the template induces the conformational change in the residues of the binding site *S*_1_, which in turn results in the conformational change in the active site that is adjacent to the residues of the binding site *S*_1_. This unpaired-base-related conformational change thus results in the active site having a much higher affinity for the structurally compatible nucleotide than structurally incompatible nucleotides. This argument is consistent with the experimental data for high-fidelity DNA Pols showing that the shape of the nascent base pair is important regardless of whether the Watson-Crick hydrogen bonds can be formed [76]. It is also consistent with the recent FRET-based assay on Klenow fragment showing that base discrimination takes place within the open complex rather than occurs during the transition from open to closed fingers conformations [77]. Conversely, the interaction of the structurally incompatible nucleotide may have a negative effect on the conformational change in the active site, which in turn results in the inverse conformational change in the binding site *S*_1_, potentially reducing its binding affinity for the unpaired base on the template. This could account for the increased dissociation caused by the binding of a mismatched nucleotide, as observed by Joyce et al. [77].

Crystal structures of the Y-family DNA Pol complexed with DNA substrate showed weak or no interaction of the Pol with the ssDNA template [27,28,32,41,42]. The weak binding affinity would result in a minor or no conformational change in the active site that is related to the structure of the unpaired base on the template, giving a smaller difference in binding affinity between correct and incorrect nucleotides. This thus results in that the Y-family Pol has a low-fidelity synthesis, which is consistent with the available experimental data [43,45]. Since minor or no conformational change in the active site occurs which is related to the shape of the template base, it is expected that the base discrimination in Y-family Pols should rely mainly on the Watson-Crick hydrogen bonding interaction, in sharp contrast to the high-fidelity replicative DNA Pols where the effect of the Watson-Crick hydrogen bonding interaction can be negligible compared to the dominant effect of the base shape. Indeed, steady state kinetic studies with incoming dNTP and DNA substrates containing difluorotoluene, which has the same shape as thymine but lacks the ability to form Watson-Crick hydrogen bonds, are poor substrates for Y-family pol η [78].

## Conclusion

In conclusion, a NBR model is proposed for the translocation of the Y-family DNA Pol along the DNA substrate, which is modified from the translocation model proposed previously for the replicative DNA Pol. The observed different features of the structures for Dpo4, Dbh and pol ι in binary and ternary forms are consistent with the NBR model. Moreover, since the interaction potential *V*_2_(*x*) for Pol η has the form of Figure 4b rather than that of Figure 3b, it is predicted that Pol η would show the translocation feature similar to Dbh and Pol ι rather than Dpo4. The obtained theoretical results on dynamic properties of the Y-family Pols by using the NBR model are consistent with the available experimental data. To further verify the model, it is hoped to test the predicted results given in Figures 8, 9, 10.

## Competing interests

The author declares that they have no competing interests.

## Authors' contributions

PX is the sole author of this paper and is responsible for developing the concepts and for writing and revising the manuscript.

## Supplementary Material

Additional file 1 **Dissociation probability *P_d1 _*during time period *T*_*p*1_**. Click here for file
