# Supplementary material for: A nucleotide binding rectification Brownian ratchet model for translocation of Y-family DNA polymerases
Source: Theor Biol Med Model. 2011 Jun 24;8:22. doi: 10.1186/1742-4682-8-22 (PMC3138451; doi:10.1186/1742-4682-8-22)
Supplement: Additional file 1 — Dissociation probability Pd1 during time period Tp1. [file 1742-4682-8-22-S1.PDF]

Supplementary Information

Dissociation probability P_{d1} during time period T_{p1}

During the time period T_{p1}, the Y-family Pol can jump between the nth site and the (n+1)th site. Thus, we consider the dissociation of the Pol along the r direction but with the Pol jumping between the two potential wells along the x direction, one with depth $E_r = E_r^{(n)} = E_n + E_0$ and the other with depth $E_r = E_r^{(n+1)} = E_{n+1} + E_0$. The ratio of the time, T_n, for the Pol to position at the nth site over the time, T_{n+1}, to position at the (n+1)th site is described by Eq. (8), which is rewritten as

$$\frac{T_n}{T_{n+1}} \approx \exp\left(\frac{E_n - E_{n+1}}{k_B T}\right). \qquad \text{(S1)}$$

The dissociation probability P_{d1} during the time period T_{p1} is thus calculated by

$$P_{d1} = T_{p1}\left(\frac{T_n}{T_n + T_{n+1}} P_d^{(n)} + \frac{T_{n+1}}{T_n + T_{n+1}} P_d^{(n+1)}\right), \qquad \text{(S2)}$$

where $T_n + T_{n+1} = T_{p1}$. $P_d^{(n)}$ and $P_d^{(n+1)}$ represent P_d given by Eqs. (3) and (4) but with $E_r = E_r^{(n)} = E_n + E_0$ and $E_r = E_r^{(n+1)} = E_{n+1} + E_0$, respectively.

Using Eqs. (3), (4), (S1) and (S2), the results of the ratio, $P_{d1} \big/ \left(T_{p1} P_d^{(n)}\right)$, versus E_n for a fixed value of E_{n+1} and different values of E_0 are shown in Fig. S1. It is noted from Fig. 6a and Fig. S1 that the dissociation probability from the two wells is sensitively dependent on the depth of the deeper well, while is insensitive to the depth of the shallower well. The dissociation probability from the two wells is 1~2-fold of that from only one well with depth equal to that of the deeper well.

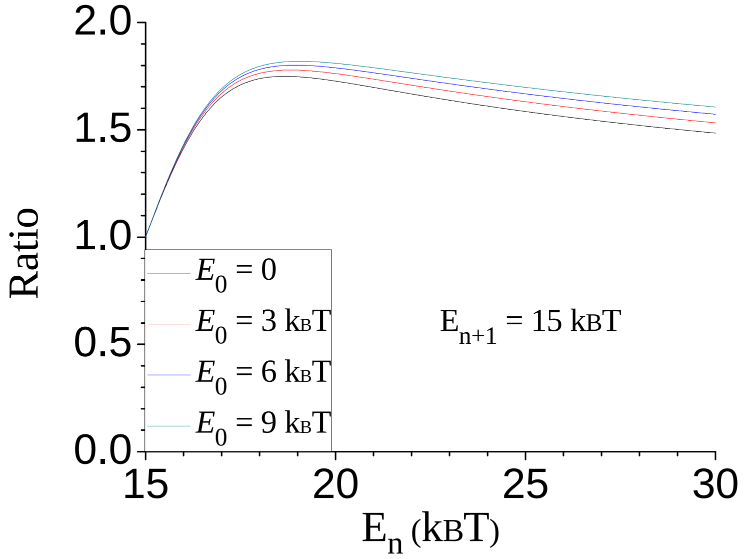

Fig. S1. Results of the ratio, $P_{d1} \big/ \left(T_{p1} P_d^{(n)} \right)$, versus E_n for different values of E_0 and a fixed value of E_{n+1}, where $E_n \geq E_{n+1}$.
